# High gas-pressure apparatus for nonlinear X-ray propagation and reshaping via stimulated X-ray Raman scattering

**DOI:** 10.1038/s41598-025-32245-x

**Published:** 2025-12-15

**Authors:** Alexander Magunia, Marc Rebholz, Tommaso Mazza, Alberto De Fanis, Thomas M. Baumann, Sergey Usenko, Nils Rennhack, Kai Li, Marc Simon, Marcus Agåker, Jan-Erik Rubensson, Michael Meyer, Linda Young, Christian Ott, Thomas Pfeifer

**Affiliations:** 1 Max-Planck-Institut für Kernphysik, 69117 Heidelberg, Germany; 2 European XFEL, 22869 Schenefeld, Germany; 3 Argonne National Laboratory, Lemont, IL 60439 USA; 4 Sorbonne Université, CNRS, 75005 Paris Cedex 05, France; 5 Department of Physics and Astronomy, Uppsala University, 75120 Uppsala, Sweden; 6 MAX IV Laboratory, Lund University, 22100 Lund, Sweden

**Keywords:** Optics and photonics, Physics

## Abstract

We describe an experimental setup for non-linear interaction and propagation of ultrashort and intense x-ray free-electron laser (XFEL) pulses in a dense gas. It allows one to provide high, but adjustable, target-gas pressures of up to 6 bar within a vacuum environment of 3∙10^− 3^ mbar or better. The setup enables investigation of intense x-ray propagation in an optically thick medium with minimal absorption loss of the unfocused beam outside of the target. As an application, we demonstrate the amplification of spectrally-resolved stimulated x-ray Raman scattering (SXRS) in dense neon gas, where the most intense inner part of the beam is almost completely absorbed. As a result, in the inner part of the beam, the SXRS signal exceeds the residual XFEL pulse by a factor of around two. In principle, this reshaping effect allows for a spatial separation of the two spectral components, i.e., the driving pulse and the SXRS signal.

**Supplementary Information:**

The online version contains supplementary material available at 10.1038/s41598-025-32245-x.

## Introduction

**Fig. 1:**
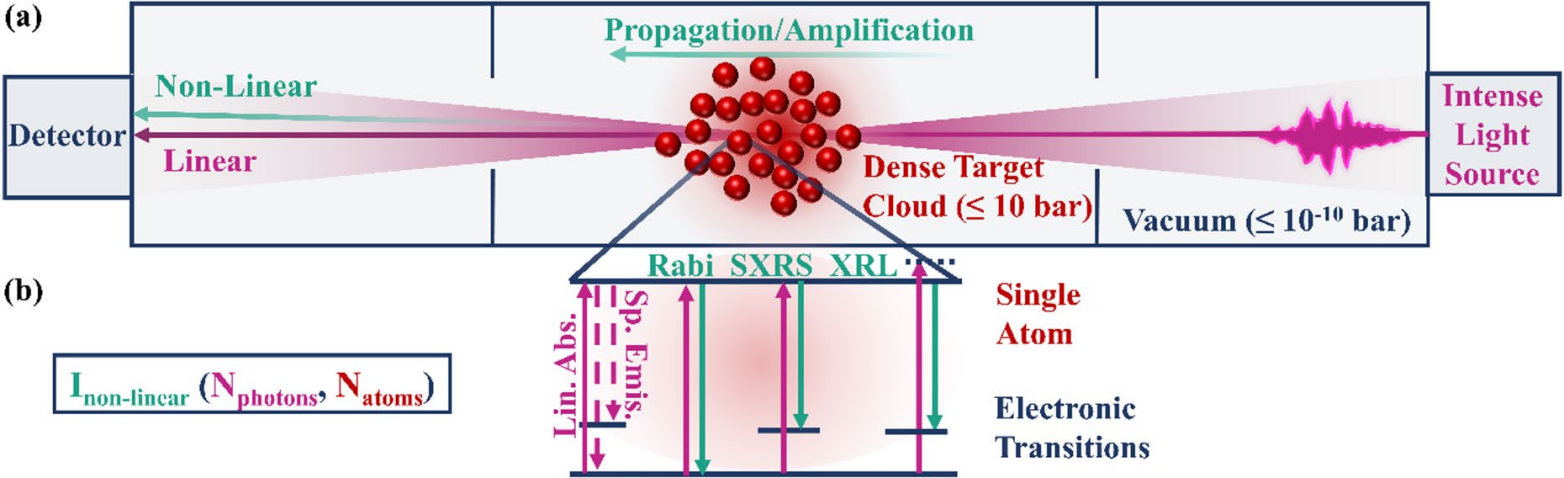
(**a**) Schematic view of the setup and physics concept of interest (propagation direction: right to left). An intense light source (pink) is focused into a dense gas cloud (red), enabling non-linear light-matter interaction, including propagation effects (green). If light is reemitted during the interaction, it can be amplified while propagating through the gas cloud. For using soft x-ray light sources, the residual setup needs to be kept in high vacuum. (**b**) Illustration of relevant (near-resonant) electronic transitions within individual atoms. Besides linear absorption, non-linear processes such as Rabi oscillations^50,51^, SXRS, or XRL take place for sufficiently high intensities during the short pulse durations. For the given examples, x-ray light can be reemitted and amplified during propagation.

The interaction of light with matter plays an important role in natural processes, including photosynthesis^1^, atmospheric radiation transfer^2^, or medical purposes such as radiation therapy^3^, and for technical devices like solar cells^4^. In many cases, the combination of the quantum-dynamics within individual atoms or molecules—described by the Schrödinger Equation (SEQ)—as well as classical wave propagation through a dense gaseous, liquid, or solid medium—described by Maxwell Equations (MWEs)—needs to be considered to understand the physical mechanisms at work^5–7^. For infrared and visible light, interacting mainly with a molecule’s internuclear degrees of freedom (e.g., vibration) or valence electrons, both micro- and macroscopic effects have been studied. Furthermore, novel extreme-ultraviolet (XUV) and soft x-ray (SXR) sources such as high-order harmonic generation (HHG)^8–10^ and free-electron lasers (FELs)^11–18^ allow for observing and steering (core-) electron motion on its natural time scale (down to the attosecond regime)^19–23^. As the XUV/SXR photon energies are sufficient for valence ionization in any material, such measurements take place under high vacuum conditions (gas pressures ≤ 10^− 7^ mbar). SXR propagation in higher-pressure absorbing media, in particular for ultrashort pulses and their resulting modified or reshaped properties^24,25^, is an emerging research field, with pioneering theoretical work^5–7,24,26–32^ and first experimental demonstrations based on HHG^25,33–36^ and FEL^37,38^ light sources. For the generation of UV, or XUV/SXR HHG pulses, setups achieving several bars of gas pressure in the interaction region have been reported based on differentially-pumped cells^39–41^, glass chips^39,42,43^ or hollow waveguides^44–46^. First propagation experiments reshaping HHG pulses^25,33–36^—which typically lack high photon numbers—are achieved in intermediate target-gas densities (below 1 bar), because the resonant dipole transitions in the XUV regime are large enough. For the SXR regime, the lower dipole transition strengths and shorter excited-state lifetimes demand larger pulse intensities for non-linear interactions and higher target pressures for propagation effects. Yet, intense SXR sources such as XFELs enable electronic processes that are only available in this regime, including x-ray lasing (XRL)^38^ and stimulated x-ray Raman scattering (SXRS)^37,47–49^. XRL describes non-resonant core-ionization, followed by the filling of the core hole in the ion with another electron, resulting in x-ray emission. In contrast, SXRS describes a resonant process in the neutral target, core-exciting and subsequently deexciting another electron by x-ray emission. This nomenclature is based on references^37,38^, whereas other x-ray communities also use the terms ‘(non-)resonant SXRS’. The emitted x-ray light leads to stimulated emission and amplification in downstream atoms, where x-ray propagation and reshaping effects are predicted^24^. These processes can require large photon numbers and target-gas densities at the same time. Yet, most experimental setups for gaseous targets at XFEL facilities deliver only intermediate target pressures below 1 bar^37^. Here, we present an experimental setup built for and operated at an XFEL facility, providing significantly higher target-gas densities (≤ 6 bar) for the non-linear interaction of ultrafast and intense SXR pulses in the dense-gas propagation regime as in^48^ (Fig. 1). The high target densities are achieved by having holes in the target-containing cell drilled by the XFEL beam, achieving a minimal gas load to the high-vacuum environment. For a proof of principle, we demonstrate saturated SXRS amplification in dense neon gas, leading to spectral and spatial reshaping of the XFEL pulses during propagation.

## Experimental setup

**Fig. 2:**
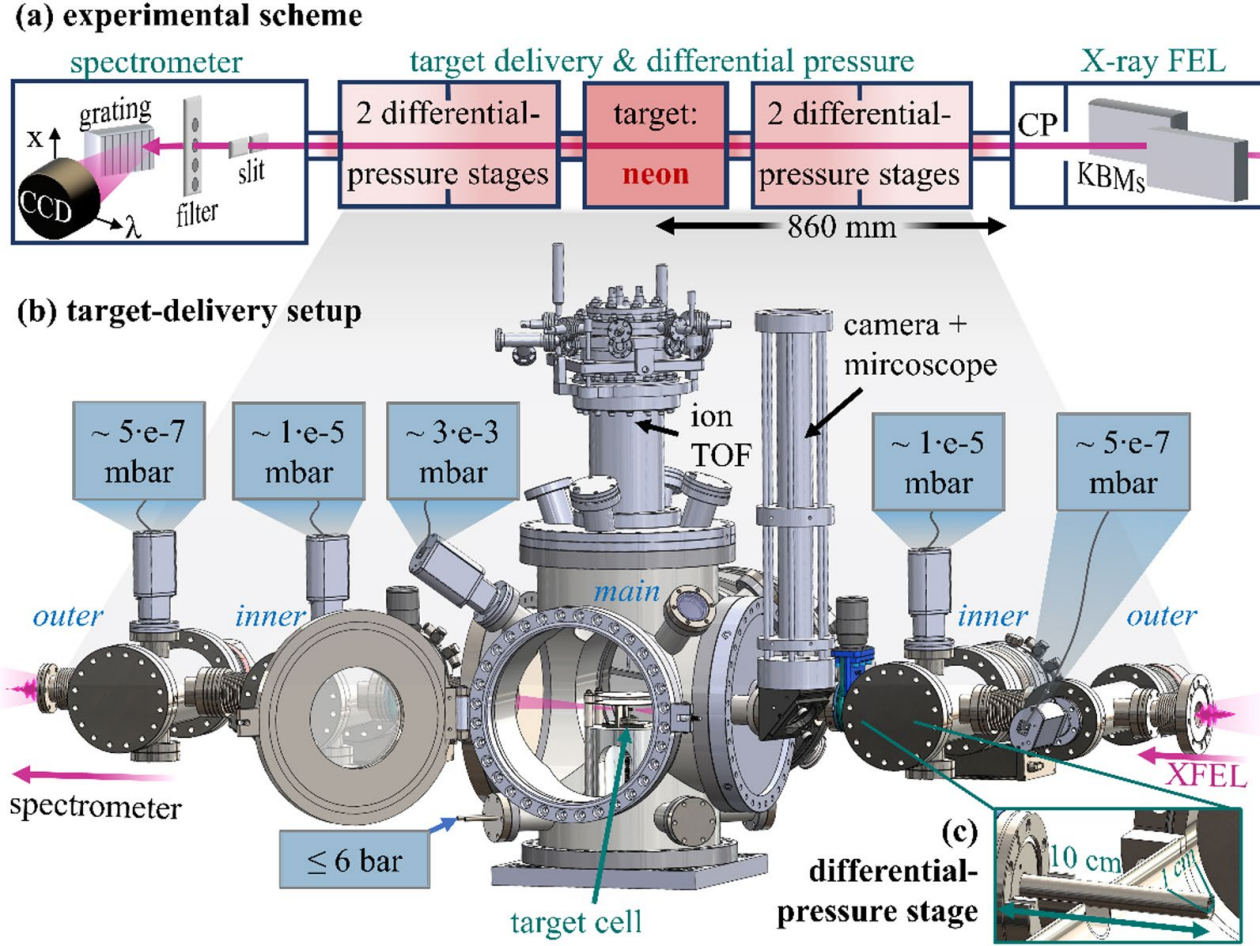
(**a**) Conceptual overview of the experiment (XFEL propagation: right to left): XFEL pulses are focused into dense neon gas and are measured with an x-ray spectrometer. KBMs = KB mirrors. CP = connection port. (**b**) 3D drawing of the target-delivery and differential-pressure setup. In the main chamber, the iTOF is placed from above, while the microscope and industrial camera are placed upstream, looking at the interaction region via a mirror. TMPs are mounted from behind. Chamber pressures (black/blue) are given for 6 bar of neon backing pressure. (**c**) Magnification of a differential-pressure chamber. The pressure reduction tubes are 10 cm long and have a 1 cm inner diameter.

The experimental apparatus is set up at the Small Quantum System (SQS) instrument at the European X-Ray Free-Electron Laser (EuXFEL) facility^16,17^. An overview of the measurement setup for the SXRS in dense neon gas is shown in Fig. 2a). A pair of Kirkpatrick–Baez (KB) mirrors focuses the XFEL pulses down to a 1–2 μm focal size at a distance of 860 mm behind the connection port of the beamline^52^. Around 1.9 m downstream of the focus, a modified *Scienta XES 350* x-ray spectrometer^53^ is used with a variable entry slit, aluminum filters, and a 5 m-radius spherical grating with 1200 lines/mm at 1.8° gracing incidence angle to disperse the x-ray light in higher-order diffraction. An x-ray sensitive *Andor Newton* charged-coupled device (CCD) camera detects the transmitted XFEL and SXRS spectra. The CCD camera has 2048$\:\times\:$512 pixels, each of size 13.5$\:\times\:$13.5 μm. The 512 pixels along the non-dispersive axis are binned to 64 pixels of effective size 108$\:\times\:$108 μm, while the dispersive axis has a resolution of 0.2 eV. The binning enables camera operation at a 10 Hz frame rate matching the XFEL repetition rate.

### Target-gas-delivery and differential-pressure setup

The target-gas-delivery and differential-pressure setup relies on a steep pressure gradient between the dense interaction region and the surrounding vacuum.

While the high target densities are contained within a small target cell, the residual setup consists mainly of vacuum chambers, turbo-molecular pumps (TMPs), and differential-pressure stages (DPSs) (Fig. 2b). The central main chamber containing the target delivery is pumped by a *Pfeiffer HiPace 2300* TMP, the two inner differential-pumping chambers have *Pfeiffer HiPace 300* TMPs attached, and the two outer chambers are pumped by *Pfeiffer HiPace 80* TMPs, respectively. At the entrance of each DPS, a 10 cm long tube with an inner diameter of 1 cm supports the pressure reduction along the beam axis (Fig. 2c). The pressures in all chambers are measured with *Pfeiffer PBR 260* gauges. The main chamber is mounted on a motorized support (*Newport*) permanently installed at SQS, which provides movement in all 6 degrees of freedom (3 translational and 3 rotational) with µm precision. Flexible bellows between the chambers allow for movement of the main chamber, while shorter bellows allow for the initial alignment of the differential-pressure chambers, which remain otherwise fixed in position. Alternatively, ion time-of-flight (iTOF) measurements^54^ can be carried out, or the focus is imaged with a fluorescent screen and a microscope industrial camera, by translational chamber movement, as described in more detail below.

### Interaction region: high-density target-gas cells

**Fig. 3:**
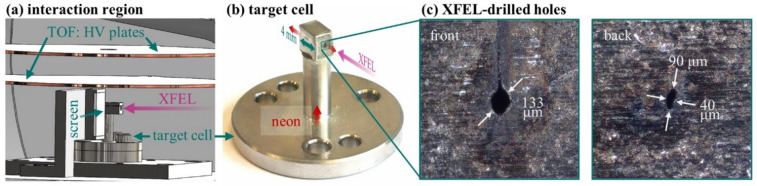
(**a**) 3D drawing of the high-pressure target cells, the fluorescent screen, and the high-voltage (HV) plates for the iTOF measurements. The upper HV plate has an extraction hole of 5 mm diameter. (**b**) Photo of the high-pressure target cell. The neon target gas (red) is supplied from the bottom and exits the cell through the XFEL-drilled holes along the beam propagation axis (pink). The target cell has an interaction length of 4 mm, and the walls containing the beam holes are 0.5 mm thick. (**c**) Microscope pictures for hole-size measurements of the XFEL-drilled cell holes after the experiment.

At the heart of the setup is the high-density target cell (Fig. 3), which is made of stainless steel and based on a well-known design used in previous x-ray absorption spectroscopy experiments^55^. Crucially, the target cell does not contain mechanically pre-drilled holes, but the tightly focused XFEL beam drills its own holes into the target-cell wall, which are 0.5 mm thick. In principle, this allows for smaller hole sizes within thicker cell walls, supporting a reduced gas flow out of the cell and thereby higher target-gas pressures. Nevertheless, the target cells are suboptimal for finding (and characterizing) the XFEL focus. Instead, by moving down the main chamber 15 mm, the XFEL focus can be brought between two iTOF high-voltage (HV) copper plates (Fig. 3a). The upper plate has an extraction hole with a 5 mm diameter, which serves as an entrance point for the generated ions to the iTOF spectrometer mounted from above. The iTOF measurements are carried out by filling the main chamber with neon gas via a second inlet at a pressure of ~ 6 ⋅ 10^− 7^ mbar, just above the background pressure of ~ 2 ⋅ 10^− 7^ mbar. The focus (1–2 μm diameter) is found by optimizing the main-chamber position and the KB mirrors for the highest count rate of highly charged ions (Ne^9+^).

Alternatively, the chamber can be moved horizontally by 7.5 mm (with respect to the target cell) to place the fluorescent screen into the highly attenuated XFEL focus or alignment laser for visual feedback. The high-density target cell can be exchanged easily, allowing usage of cells of different lengths, for which the main chamber can be sealed off with gate valves from the residual vacuum setup. For the first experiments, a cell with 4 mm inner diameter along the beam axis and 0.5 mm thick walls is used (Fig. 3b). The holes are created with a short burst of XFEL pulses, as documented in the video in the supplementary material. While the lower limit for the drilled cell holes is on the order of the XFEL focus, they are expected to grow larger in size with time due to XFEL pointing fluctuations and plasma effects in the neon gas. After one week of experiment, the roughly circular front hole was measured to be 133 μm in diameter and the elliptical back hole was measured to be 90 μm x 40 μm in size (Fig. 3c). The growth of the cell holes after their initial creation can be tracked by measuring the pressure in the surrounding main chamber, as discussed in the following.

### Gas-flow and pressure-gradient characterization

**Fig. 4:**
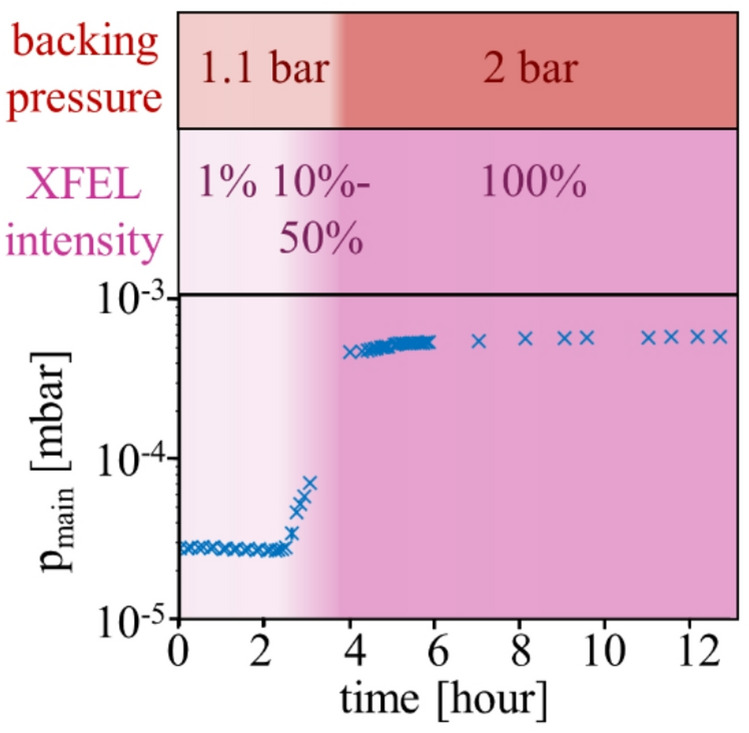
Main-chamber pressure *p*_*main*_ for the first 12 h after the cell-hole creation. In that time, the XFEL intensity is increased from 1% to 100%, and the backing pressure changes from 1.1 bar to 2 bar. For low intensities (0 h < t < 2.5 h), *p*_*main*_ remains constant, implying a constant XFEL-drilled hole size. With increasing intensities from 10% to 50% (2.5 h < t < 3.5 h), *p*_*main*_ increases, implying an increase in the target hole sizes. For maximal intensity (t > 4 h), *p*_*main*_ stabilizes within the first two hours and then remains constant, implying no further increase in hole size.

The main-chamber pressure is tracked for the first 12 h after the creation of the target-cell holes for various pulse intensities and target backing pressures (Fig. 4). For the first 2.5 h, the XFEL pulses (at ~ 867 eV photon energy) are attenuated down to 1% of the maximal pulse energy (~ 6 mJ) by using the SASE3 gas attenuator^56^. The backing pressure of the neon target gas is set to a minimal value of 1.1 bar with the gas-flow regulating *Festo* controller. In this period, the main-chamber pressure stays constant around ≤ 2 ⋅ 10^− 5^ mbar, implying a constant target-cell hole size. After increasing the pulse intensity within the next hour to 50% in 10% steps (with respect to the unattenuated beam) for unchanged backing pressure, the main-chamber pressure increases up to ≤ 8 ⋅ 10^− 5^ mbar, indicating a widening of the XFEL-drilled cell holes. For an unattenuated XFEL beam (100%), the main-chamber pressure has only been recorded for a simultaneous increase of the neon backing pressure to 2 bar. Yet, within the first two hours of the target cell being exposed to maximum XFEL intensity, the main-chamber pressure stabilizes at ≤ 6 ⋅ 10^− 4^ mbar, where it stays constant for unchanged pulse and target settings for the next 6 h. Combining experimental information from Figs. 3 and 4, we conclude that stable conditions for the one week of experiment are reached within 2 h, such that the target cell holes are already much larger than the XFEL focus diameter. Hence, no truncation or diffraction of the XFEL beam is expected for the later experiments.

**Fig. 5:**
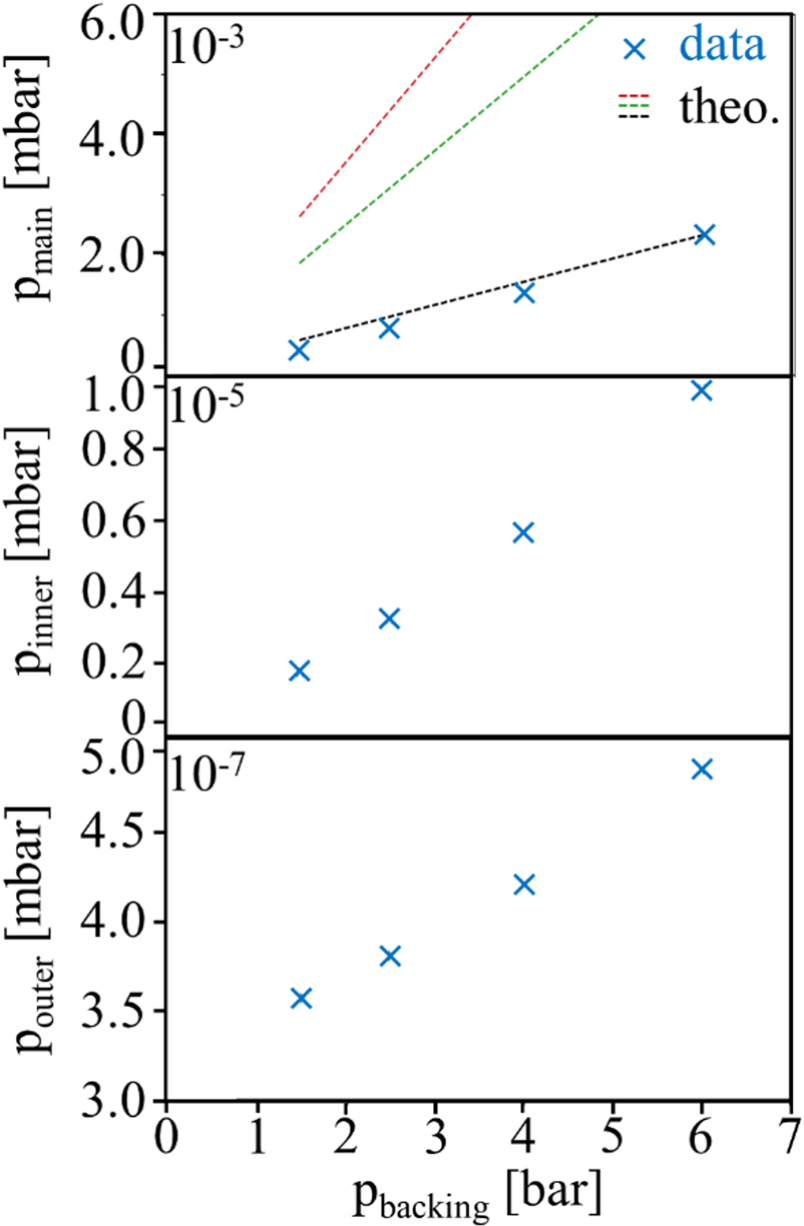
Chamber pressures as a function of backing pressure of the neon target gas. Measured chamber pressures for the (**a**) main, (**b**) inner, (**c**) outer chambers are represented with blue crosses. The error bars estimated by averaging over half an hour of measurement are too small to be visible on the shown scale. The results for the main-chamber pressure of the three theory models (Table 1) are shown with dashed lines, black: (I) molecular-tube model, green: (III) molecular-aperture, and red: (IV) viscous aperture.

Further, once the target-cell hole sizes and main-chamber pressures have stabilized, target-pressure-dependent experiments can be performed. For the high-pressure region, the *Festo* controller can be adjusted from 1.1 bar to 11 bar in steps of 0.1 bar. Additionally, a leak valve (*Pfeiffer EVR 116*) can be continuously closed to further decrease the backing pressure in the range of ~ 10 mbar to 1 bar, allowing for target pressures typically used in transient absorption spectroscopy experiments without propagation effects. The gas flow through the target-delivery and pressure-gradient setup is characterized by measuring the pressures of the main, inner, and outer chambers for different backing pressures of the target neon gas in the range from 1.5 bar to 6 bar (Fig. 5). All three chamber pressures show a linear dependence on the backing pressure. Based on the hole sizes estimated with the microscope (Fig. 3), the conductance of the target cell can be estimated based on four different models assuming molecular or viscous flow through aperture- or tube-like cell holes^57^, shown in Table 1.

**Table 1:** Conductance for different assumptions of flow regimes and cell-hole geometries^57^. Here, $\:\stackrel{-}{\text{v}}$ = 555 m/s is the average velocity of the neon atoms at room temperature, and D and L are the inner diameter and length of the connecting target-cell holes (in cm), $\:{\upeta\:}$ = 297 µP is the viscosity of neon, P_av_ is the average pressure inside the cell hole, T = 295 K is the temperature of the neon gas, and M = 3.35$\cdot$10^−26^ kg (20.18 u) is the molecular mass of the neon atoms.

Flow type	Cell-hole type	Conductance [in l/s]	Model
Molecular	Tube	$\:C=2.6\cdot\:{10}^{-4}\:\stackrel{-}{v}\:\frac{{D}^{3}}{L}$	(I)
Viscous	Tube	$\:C=\text{32,600}\cdot\:{P}_{av}\cdot\:\frac{{D}^{4}}{\eta\:L}$	(II)
Molecular	Aperture	$\:C=5\pi\:\cdot\:{D}^{2}$	(III)
Viscous	Aperture	$\:C=\frac{3.7}{4}\pi\:\sqrt{\frac{T}{M}}\cdot\:{D}^{2}$	(IV)

The total conductance of the two target-cell holes is the sum of the individual hole conductances. Together with the pumping speed *S*_*p*_ of the main-chamber TMP for neon gas, which is 1900 l/s, the pressure *p*_*main*_ after the connecting tube- or aperture-like hole cells can be expressed as a linear function of input cell pressure *p*_*cell*_^57^:

1 $$\:{p}_{main}=\:\frac{1}{1+\frac{{S}_{p}}{C}}\cdot\:{p}_{cell}.$$

For a pressure-independent conductance, as in models (I), (III), and (IV), the main-chamber pressure is a linear function of the input pressure. Yet, for (II), this is not the case, which contradicts the experimental findings and will not be further considered. For the other three cases, the resulting main-chamber pressures as a function of backing pressure are shown in Fig. 5a) as dashed lines in black (I), green (III), and red (IV). As a result, model (I), a tube-like cell hole in the molecular-flow regime, is the best-fitting model, being in excellent agreement with the experimental data. It should be noted that a pressure reduction between the backing pressure and cell pressure has not been considered.

## Results: stimulated X-ray Raman scattering (SXRS) in dense neon gas

The process of (resonant) SXRS was demonstrated experimentally about a decade ago in neon gas^37^, where a photon of 867 eV photon energy is absorbed to promote an electron in neon from the 1s state to the 3p state, which is accompanied by the deexcitation of a 2p electron into the just-created 1s core hole by emitting a photon of 849 eV photon energy. At sufficiently high x-ray photon flux and neon density, the emitted photon then drives the stimulated version of the process along the residual gas cloud. By using photon-recoil imaging, SXRS can also be detected in the single-particle regime^49^. There, atomic momenta instead of the re-emitted photons are measured. The nearly identical, but incoherent process, known as resonant inelastic x-ray scattering (RIXS)^58^ is based on purely spontaneous emission of the scattered photons, which happens at low photon flux and target density as well. It has been applied to a wide range of targets, such as gas-phase atoms or molecules, liquids, and transition-metal complexes^59^. Yet, since the pioneering discovery of SXRS in gas-phase neon, performing SXRS on molecular or condensed-matter targets remained elusive, as the SXRS cross sections are much smaller^60^. The situation changed with a recent experiment in solid Co/Pd multilayer samples^61^, as well as the introduction of impulsive stimulated x-ray Raman scattering (ISXRS), which uses a spectral wing of the driving pulse to stimulate the photon emission^62^. ISXRS has been demonstrated successfully in gas and liquid-phase molecular targets^47,62^. Nevertheless, improvements in experimental capabilities for any of the SXRS versions are desirable, as demonstrated by super-resolution SXRS^48^. To this end, we have reinvestigated the pioneering work in neon^37^ with the new capabilities of the setup described above.

**Fig. 6:**
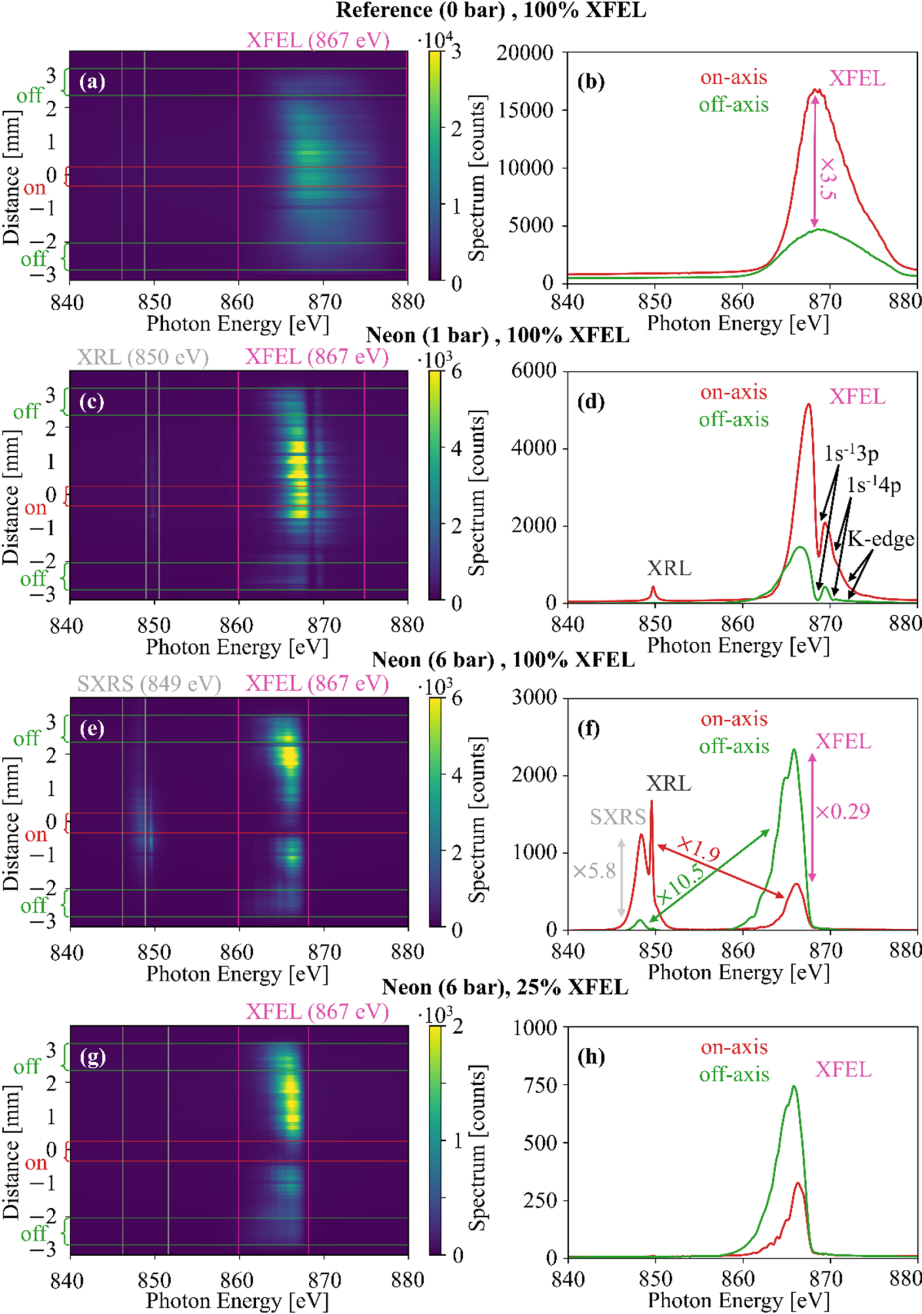
Measured XFEL transmission spectra for various target-gas pressures (0 bar, 1 bar, 6 bar) and XFEL pulse energies (25%, 100%). Left: (**a**,**c**,**e**,**g**) Spatially and spectrally resolved spectra. Right: (**b**,**d**,**f**,**h**) Spatially averaged spectra for on- and off-axis regions, as marked with red/green lines in the left-side plots. (**a**,**b**) Reference spectra without the target gas. The spectral wings at the higher-energy side reach close to 880 eV. Spatially, the on-axis part is 3.5 times more intense than the off-axis part. (**c**,**d**) Transmission spectra with 1 bar of neon pressure, and at full pulse energy. High-energy parts of the XFEL spectrum are absorbed by the 1s^− 1^np Rydberg series (867-870 eV) and K-edge ionization continuum (> 870 eV). Only a small XRL signal is evident. (**e**,**f**) Same as (c, d), but with 6 bar of neon pressure. Significant spectro-spatial reshaping of the XFEL pulse occurs, including an SXRS signal. (**g**,**h**) Same as (e, f), but at 25% pulse energy. Absorption, but no emission is evident.

To obtain a reference for the later introduced spectro-spatial reshaping, we measure, without target gas, the XFEL spectrum centered at 867 eV, which is spatially resolved perpendicular to the dispersive axis (Fig. 6a). The spatially averaged part in the ‘on-axis’ region (at ~ 0 mm, red) is larger than with respect to the spatial ‘off-axis’ regions (at $\:\pm\:$3 mm, green) (Fig. 6b).

**Fig. 7:**
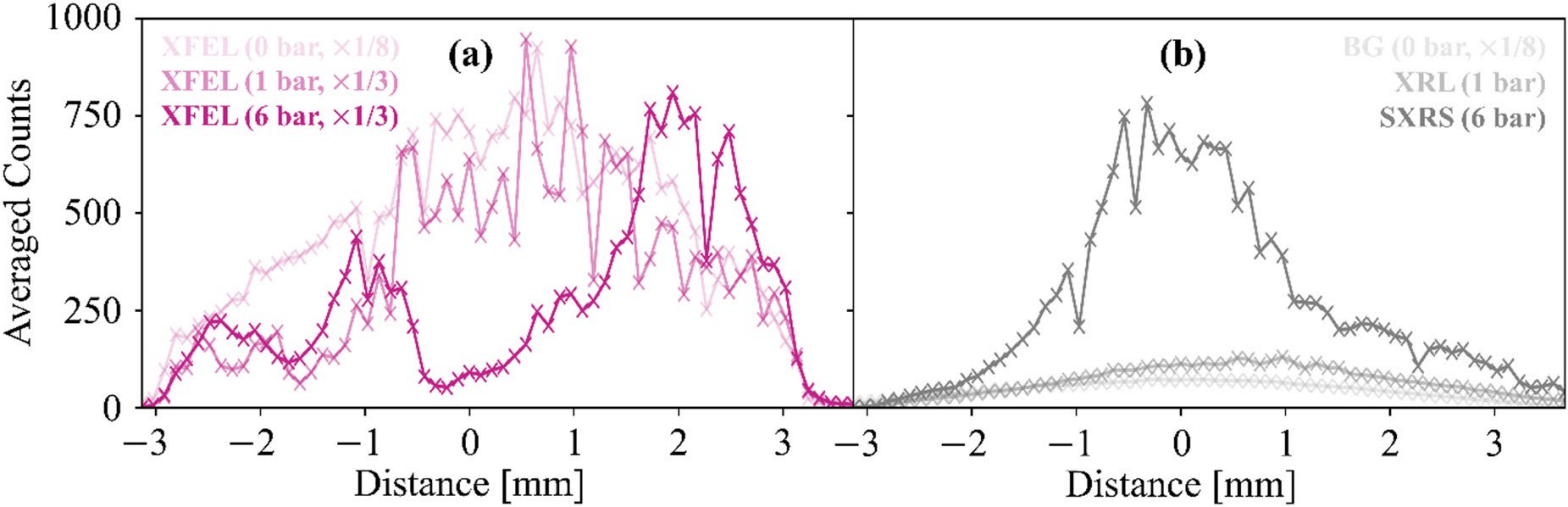
XFEL and SXRS spectrally averaged along the non-dispersive axis. (**a**) Averaged XFEL, and (**b**) averaged SXRS signals for 0 bar, 1 bar, and 6 bar of neon-target-gas backing pressure. As given in the legend, certain lineouts are multiplied by a factor of 1/3 or 1/8 to enable an easier qualitative comparison between lineouts.

In the previous work^37^, the most intense XRL and SXRS emission coincides with the intense inner part of the driving XFEL pulse. To reproduce this behavior, we measure at 1 bar of neon backing pressure (comparable to 0.7 bar in^37^), and the full XFEL pulse energy of 6 mJ (around one order of magnitude higher than in^37^, Fig. 6c, d). Part of the XFEL spectrum is absorbed by the 1s^− 1^np Rydberg series (for photon energies between 867 eV and 870 eV) and the K-edge ionization continuum (for photon energies above 870 eV). Also, a weak XRL signal is visible at 850 eV, which smoothly follows the intensity of the XFEL pulse along the non-dispersive axis (see also Fig. 7). In contrast to the previous work^37^, we do not see any SXRS signal. We attribute this to a combination of factors, such as much higher pulse energy, and likely different spectral distribution with respect to the configuration in^37^, leading to the XRL dominating over the SXRS process in this very specific configuration, potentially also because of reabsorption of the SXRS emission. A more detailed explanation requires an in-depth theoretical investigation, which is beyond the scope of this work.

Furthermore, we demonstrate a new regime of SXRS, where we drive the amplification process hard enough by using 6 bar of neon backing pressure and 6 mJ of XFEL pulse energy (Fig. 6e, f). Here, the transmitted driving pulse has nearly vanishing intensity (Fig. 6e), in the ‘on-axis’ center of the non-dispersive axis, but a residual spectrum in the ‘off-axis’ regions. At the same time, strong SXRS and XRL signals are created at 849 and 850 eV, appearing close to the center of the non-dispersive axis. This implies that most incoming XFEL photons are absorbed in the dense gas cloud, and a significant portion is re-emitted via XRL/SXRS. As the process is more likely for (i) high gas densities and for (ii) high driving pulse intensities, this was not observed in previous work^37^. In our results (Fig. 6), this effect is only observed ‘on-axis’ where the incoming XFEL intensity is sufficiently high for driving the non-sequential SXRS. This is further illustrated with the comparison of spatially averaged lineouts between the ‘on-/off-axis’ regions (Fig. 6f). The ‘on-axis’ XFEL spectrum is less intense than the ‘off-axis’ part (by a factor of 0.29, pink arrow). At the same time, the ‘on-axis’ SXRS signal is 5.8 times stronger than the ‘off-axis’ SXRS signal (grey arrow). Critically, the ‘on-axis’ SXRS signal is 1.9 times more intense than the transmitted ‘on-axis’ XFEL spectrum (red arrow), whereas the ‘off-axis’ XFEL spectrum is 10.5 times more intense than the ‘off-axis’ SXRS signal (green arrow).

To illustrate to complexity of this x-ray propagation effect, we repeat the measurement at 6 bar of neon backing pressure, but with 25% of the pulse energy, i.e., 1.5 mJ (Fig. 6g, h). The transmitted XFEL spectrum around 867 eV is nearly identical to the one at 6 mJ (Fig. 6e, f), but no XRL/SXRS signal can be detected at 849 eV. This implies a regime where the XRL/SXRS processes are highly nonlinear with respect to the driving XFEL intensity (and not just with the target gas density). While not a direct experimental observation, one can regard this as an indirect sign that more complex spectro-spatial reshaping effects, such as self-focusing, are taking place. They are predicted by theory in^24^, but no spectrally resolved transmission spectra are computed in that work for a direct comparison to our findings. As in the case of 1 bar backing pressure, where only an XRL without an SXRS signal appears, the required theory is beyond the scope of this work.

To highlight a potential usage of this nonlinear regime, the XFEL and SXRS signals are spectrally averaged within the violet and grey vertical lines in Fig. 6a, c, e), and are shown as a function of the non-dispersive axis in Fig. 7. In the case of 1 bar backing pressure, the transmitted XFEL spectrum is only slightly modified with respect to the reference XFEL spectrum measured without target (Fig. 7a). At the same time, the XRL signal smoothly follows the intensity of the XFEL pulse along the non-dispersive axis, but it is only slightly above the background measured without target gas (Fig. 7b). In contrast, for the 6 bar backing pressure, the transmitted XFEL pulse has a minimum at 0 mm on the non-dispersive axis, where the XFEL reference has its maximum (Fig. 7a). Yet, the SXRS signal generated in the dense target has a maximum at 0 mm. This maximum is as broad as the corresponding XFEL minimum (± 1 mm), but appears narrower along the non-dispersive axis compared to the initial XFEL spectral distribution (± 2 mm).

In principle, this allows for a spatial separation of the driving XFEL pulses centered at 867 eV and the SXRS signal centered at 849 eV. Furthermore, it has also been demonstrated that temporal reshaping of the XFEL pulses occurs during the XRL/SXRS processes^24,51^, which could be investigated experimentally in the future, e.g., by looking at the ‘on-/off-axis’ regions separately by placing appropriate slits across the far-field beam.

## Conclusion

In summary, we have presented an experimental gas-target setup operating at high pressures—6 bar of neon—and at a high-intensity XFEL-pulse facility (EuXFEL) to drive the process of stimulated x-ray Raman scattering (SXRS) and X-Ray Lasing (XRL). As an application, we identify two spatial regimes: (i) off-axis, where the transmitted XFEL spectrum is dominant with respect to the induced SXRS signal; (ii) on-axis, where the SXRS is predominant compared to the residual XFEL spectrum. In addition, we have identified experimental conditions (867 eV photon energy, 6 mJ pulse energy, 1 bar backing pressure), for which the XRL dominates the SXRS process.

Furthermore, we have demonstrated that the setup is capable of operating with high target density for a typical beamtime period of one week, all while maintaining ultra-high vacuum outside of the main experimental chamber required by sensitive beamline equipment. We conclude that the novel approach of XFEL-self-drilled holes within the target cell is sufficiently reliable and stable to perform an experimental campaign lasting one week, i.e., the default duration of FEL beamtimes. At the same time, this approach also overcomes any potential alignment or cell-damaging problems, which can occur when using pre-drilled holes.

In the future, the ratios between XRL and SXRS signals, as well as the transmitted XFEL spectrum in the ‘on-/off-axis’ regions, can be further optimized by using different target-cell lengths (along the propagation axis) and adjusted target-gas pressures or XFEL pulse energies. By using different target-cell materials, different wall thicknesses, or nested-cell and differential-pumping designs, it should be possible to achieve even higher target-gas pressures. This and future work can provide the experimental basis for detailed theoretical analysis, enabling a precise understanding and optimization of the underlying propagation.

From a laser-engineering perspective, one could consider this approach for spectral, spatial, and temporal reshaping of the XFEL pulses and reusing the reshaped XFEL pulses in a different target medium. From a scientific perspective, SXRS is an excellent spectroscopic tool with femtosecond time resolution, which can be used to study many other target gases, both atomic and eventually molecular. This tool is now available with extremely high resolution, e.g., resolving Rydberg states and their fine-structure splitting—which we showed in^48^, using the same setup as described here—and at unprecedented amplification levels of the SXRS signal, as shown in this work. This could be used to investigate, for example, site-specific electron dynamics in molecules, and resulting dissociation pathways, e.g., improving on our findings in CF_4_^63^—which are also based on the here reported setup. The high SXRS amplification gains could be beneficial to overcome the previous limitations of low SXRS cross sections in molecular targets^60^.

## Electronic Supplementary Material

Below is the link to the electronic supplementary material.

Supplementary Material

## Data Availability

The experimental data were collected during the beamtime p2748 at EuXFEL. Raw and meta-data are available at: https:/in.xfel.eu/metadata/doi/10.22003/XFEL.EU-DATA-002748-00.
